# Carbon nanomaterial Formation on Fresh-Reduced Iron by Converted Natural Gas

**DOI:** 10.1186/s11671-017-1882-6

**Published:** 2017-02-10

**Authors:** A. Nebesnyi, V. Kotov, A. Sviatenko, D. Filonenko, A. Khovavko, B. Bondarenko

**Affiliations:** Gas Institute of the NAS of Ukraine, Degtyarivska Str., 39, 03113 Kyiv, Ukraine

**Keywords:** Carbon monoxide, Fresh-reduced iron, Carbon cuff, Iron fragmentation, Carbon nanotubes

## Abstract

The mechanism of carbon nanomaterial formation at moderate temperatures while processing fresh-reduced iron by products of air conversion of natural gas is considered. It is shown that under given conditions, the size and the shape of the resulting carbon are dependent on the temperature and the size of microscopic iron grains formed during reduction. These iron grains are the catalyzer of the reaction of carbon monoxide disproportionation. It is concluded that the formation of a nucleus of the new carbon phase occurs at the contact boundaries of neighboring grains of newly reduced iron with the subsequent formation in these places of ring-shaped carbon cuffs. Nanotubes are forming as a result of further carbon crystallization, and separation of iron particles from the main mass is occurring, i.e., there is a fragmentation of the substance of the catalyst. According to the results of laboratory studies, the optimum temperature of carbon nanotube formation in the environment of converted gas is 600–650 °C. The evidence of the hypothesis that the mechanism of the reaction of carbon monoxide disproportionation flows through the intermediate stage of iron oxides formation is given.

## Background

Recirculated throat (waste) gases are typically used for cooling the product produced in shaft furnaces of direct iron reduction [1]. In these circumstances, the process of carbon formation is released. This carbon is formed from carbon monoxide contained in the waste gases. It is known [2] that the fresh-reduced iron is a catalyst in the decomposition reaction of carbon monoxide, while its decomposition in a pure gas environment at atmospheric pressure is practically not observed.

Currently, there is no generally accepted view on the mechanism of carbon monoxide formation, including the formation of carbon nanomaterials. Previously, it was widely believed that adsorption-catalytic theory of the dissociation lies in the basis of the mechanism of the process of carbon release from its monoxide [3, 4]. According to this theory, a significant weakening of the link between atoms of carbon and oxygen occurs due to the adsorption of molecules of carbon monoxide on the iron surface, which upon subsequent collision of a new molecule of CO with an adsorbed molecule, facilitates the destruction of this bond according to the reaction

1 $$ {\mathrm{CO}}_{\mathrm{g}}+{\mathrm{CO}}_{\mathrm{ads}.}={\mathrm{CO}}_2+\mathrm{C}. $$

However, further studies showed that while chemisorption of the CO molecule on the catalyst occurs, weakening of the bond between carbon and oxygen atoms occurs very slowly. This fact suggests that the mechanism of carbon formation is unlikely [5].

It is suggested [6] that carbon formation by decomposition of hydrocarbons can run through the stage of iron carbide formation on the catalyst, followed by its decay according to the reaction

2 $$ {\mathrm{Fe}}_3\mathrm{C}\to 3\mathrm{F}\mathrm{e}+\mathrm{C}. $$

Carbon may be formed, depending on the conditions, widely differing between each other flake form or in the form of carbon nanotubes, nanofibers, and in a special conditions, in a form of fullerenes [7] or diamonds [8].

## Methods

Below are results presented from the research and analysis of carbon material formation on an iron catalyst in the by-products of air conversion of natural gas. Steel plate of Steel 3 served as a substrate for obtaining the catalyst. Iron oxide (III) has been formed on these plates followed by its reduction by hydrogen in a quartz reactor. After the iron reduction reactor has been blown by nitrogen, products of air conversion of natural gas, dried on the silica gel, were forwarded into the reactor. Products of the conversion were of the following composition (%, vol.):

$$ \mathrm{C}\mathrm{O}=18{\textstyle \hbox{-} }19;{\mathrm{H}}_2=32{\textstyle \hbox{-} }35;{\mathrm{CO}}_2=1,5{\textstyle \hbox{-} }3;{\mathrm{CH}}_4=0,2{\textstyle \hbox{-} }0,5;{\mathrm{N}}_2{\textstyle \hbox{-}}\mathrm{therest}. $$

**Fig. 1:**
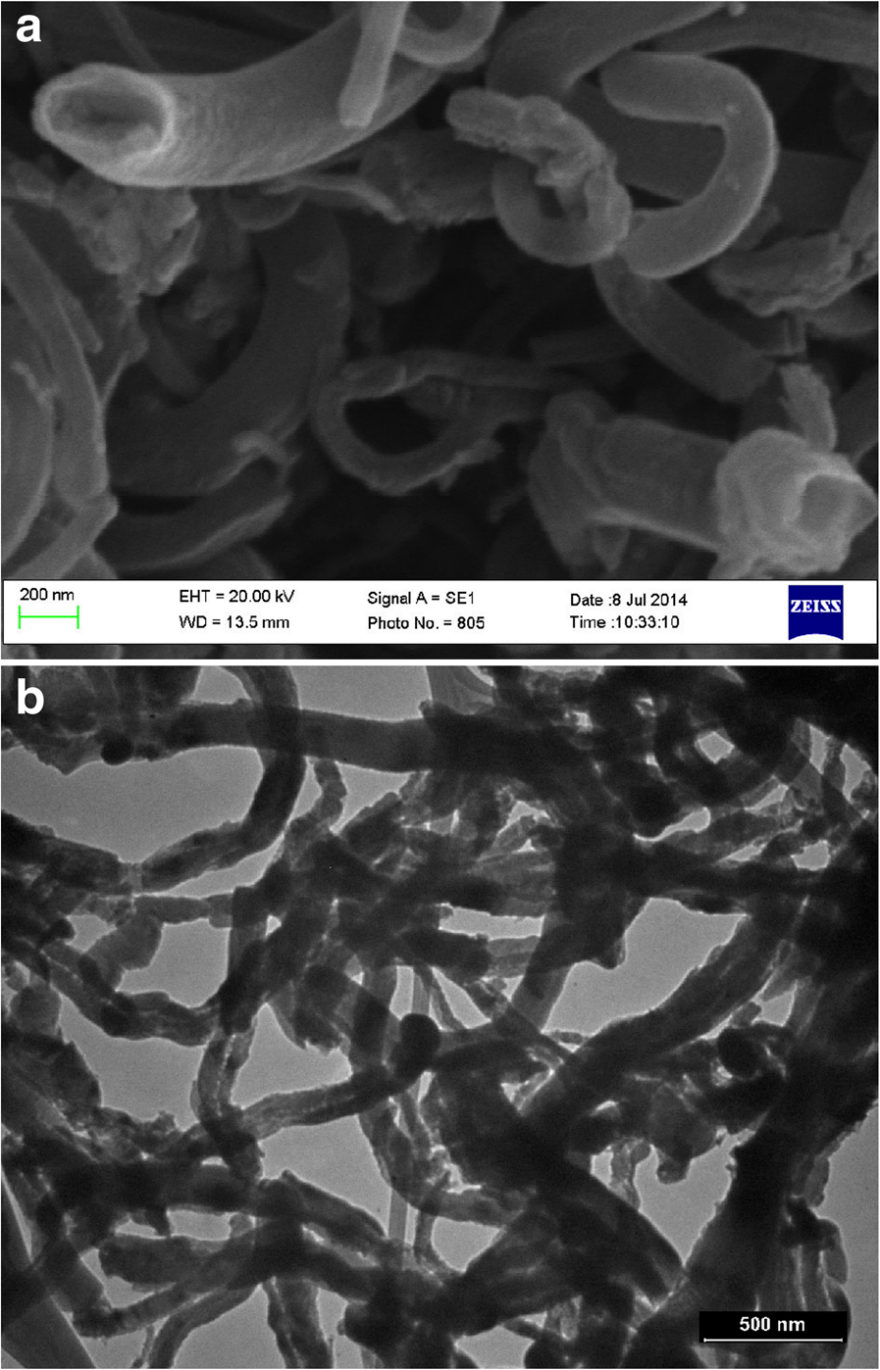
Photo of the carbon material obtained as a result of fresh-reduced iron processing by converted natural gas (*t* = 650 °C)

The study of the obtained carbon material was performed by using the following electron scanning microscopes: JEOL JSM-6700 F and ZEISS EVO 50 XVP. Translucent photomicrographs of samples of the material obtained from the iron catalyst show that the material consists of multi-walled carbon nanotubes (CNTs) with an outer diameter of up to 300 nm (Fig. 1). These “colossal” nanotubes were also obtained as a result of the pyrolysis of the propane-butane mixture in a plasma [9].

## Results and Discussion

Based on the analysis of literature and data of laboratory studies [10, 11], the mechanism of CNTs formation in the area of moderate temperature (up to 700 °C) in a general form can be represented as follows.

Volume of metal oxide decreases at the process of its reduction. This fact can be estimated by the expression:

3 $$ {V}_{\mathrm{oxide}}/{V}_{\mathrm{metal}}=\left({M}_{\mathrm{oxide}}/{\rho}_{\mathrm{oxide}}\right)/\left( n\cdot {A}_{\mathrm{metal}}/{\rho}_{\mathrm{metal}}\right), $$

where *V*_oxide_ and *V*_metal_ are, accordingly, the volumes of oxide and metal formed from it after reduction; *A*_metal_ and *M*_oxide_ are the atomic weight of the metal and the molecular weight of its oxide; *ρ*_metal_ and *ρ*_oxide_ are the density of the metal and its oxide; and *n* is the number of metal atoms in the molecule of its oxide.

In the case of Fe_2_O_3_ reduction, we have *M*_oxide_ = 160; *A*_Fe_ = 56; *n* = 2; *ρ*_Fe_ = 7.9; *ρ*_Fe2O3_ = 5.1, then after substituting these data in expression (3), we get

3a $$ {V}_{\mathrm{Fe}2\mathrm{O}3}/{V}_{\mathrm{Fe}}=2.2, $$

i.e., the volume of iron (III) oxide at its reduction becomes smaller more than two times. A highly dispersed structure of iron in the form of chaotic piles of separate grains is formed as a result of restructuring of the crystal lattice and decrease of the catalyst volume. Further, this phenomenon has a considerable influence on the formation of the resulting carbon material.

**Fig. 2:**
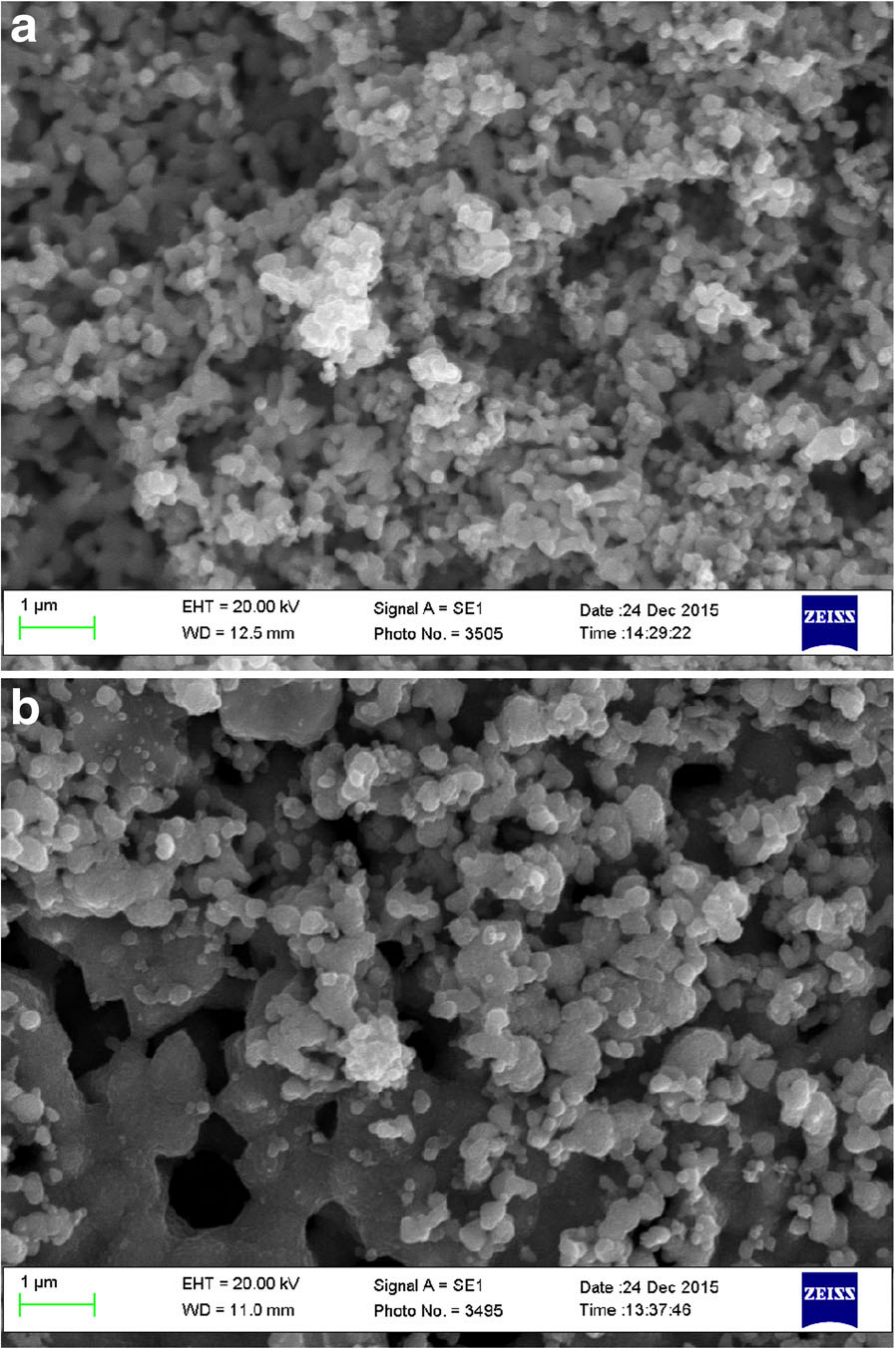
Photo of the samples surface of the iron catalyst after reduction by hydrogen at a temperature of 450 (**a**) and 650 °C (**b**)

The effect of the temperature of the catalyst reduction on the size of the generating iron particles are shown in Fig. 2. As follows from the figure, the size of the resulting grains of iron decreases with decreasing temperature, while the mobility (self-diffusion) of atoms (ions) and, consequently, the process of collective recrystallization and consolidation of the particles slows down sharply.

As a result, the material gets a developed surface [12] and consists of small variously oriented crystals of iron, a lattice of which contains many defects. Iron atoms have increased activity because of noncompensated chemical bonds, a manifestation of iron ignitibility. Boundaries are formed of a type of “insular Motta model” between touching grains of the metal [13]. Order in the arrangement of atoms could largely be absent on the boundary of grain contact according to this model. Such boundaries are called incoherent.

It is known [14] that the largest number of defects in the crystal lattice of iron is on incoherent boundaries of contact of the grains, contacting with each other. These areas have high catalytic activity. The release of carbon atoms first of all occurs at the process of CO decomposition with a subsequent formation of nuclei of a new solid phase. The emergence of a new phase at the grain boundaries also contributes to a higher diffusion rate of carbon atoms in these locations [15, 16]. Located on the surface of the crystal lattice of the iron atoms, in contrast to that which is situated inside due to noncompensated chemical bonds between them, a force field is created [17]. The effect of these fields is enhanced at the points of contact of contiguous iron grains. They presumably also contribute to the catalytic activity of these places and the emergence of a new phase.

Prior to carbon phase formation, the induction period exists during which there is an accumulation of free carbon atoms with the increase of their concentration up to a critical value at which the beginning of a new phase formation occurs. Study on thermogravimetric installation shows that the induction period at a temperature of 650 °C is about 8–10 min.

**Fig. 3:**
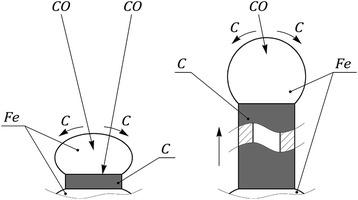
**a** Scheme of carbon cuff formation on the contact border of the catalyst grains. **b** Growth of CNTs after iron particle separation from the main part of the catalyst

A new phase appearing at the points of contact of adjacent grains of iron catalyst initially has the form of a ring-shaped carbon cuff Fig. 3a. The similar phenomenon is observed, for example, at process of the pelletizing of moist fine material, when the water cuffs are formed in places of particle contact [18]. The formation of a carbon cuff is like the formation of a water cuff which further contributes to the preferential formation as not solid, but hollow fibers, i.e., nanotubes.

The resulting carbon cuff provides for a wedging effect on grains of a metal, and as a result of a subsequent deposition (crystallization) of carbon atoms (at constant outer and inner diameters of the cuff), the separation of grains from the main “mainland” part of the catalyst occurs (Fig. 3b). Thus, the fragmentation of the grains of iron at the initial stage of the formation of carbon nanotubes occurs after dispersion of the catalyst metal at the phase of metal reduction. It should be noted that stresses occurring during carbon crystallization can reach large values. As the results show cases of destruction of iron ore pellets, masonry of blast furnaces in the environment of CO-containing gases have a place in practice.

The atoms of carbon formed on various active centers of the surface of a separated catalyst particle, due to the existing concentration gradient, diffuse toward the board of contact of the particles with the nanotube, where their crystallization occurs. The growth of nanotube primarily oriented along the line connecting the centers of contacting catalyst grains. Because grains are mainly randomly located, growth of nanotubes occurs in different directions relative to the wafer surface coated by a catalyst. In addition, the catalyst activity in different areas of the separated iron particle is not identical and therefore carbon deposition rate at different points along the line of particle contact with the growing nanotube varies; this leads to curvature of the latter. Apparently, mechanical interaction of the tubes with each other at the process of their growth also contributes to curvature. As a result, the material looks like “tangled hair.”

**Fig. 4:**
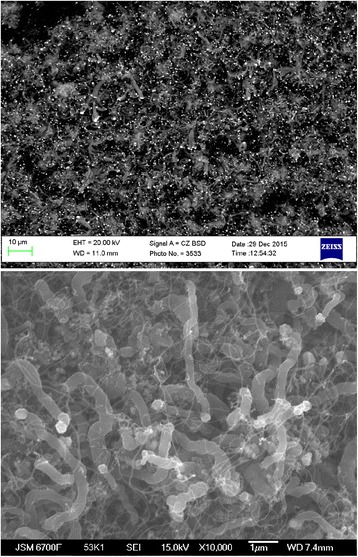
Photo of CNTs samples illustrating: **a** Fragmentation of iron catalyst, iron particles (*light dots*). **b** The conformity of the outer diameter of the formed CNTs to the size of separated iron particles

The presence of iron particles at the ends of forming nanotubes (Fig. 4a) may serve as an indirect confirmation of the fact that boundaries of contact of the catalyst grains to each other are the initial allocation of carbon and the formation of a new phase. It is noteworthy that the particle size of the iron at the ends of the carbon nanotubes corresponds to the outer diameter of these tubes. This is clearly seen on photomicrographs (Fig. 4b), which show two arrays of CNTs having a tube diameter of about 300 and 70 nm; iron particles located on their ends have the same size. Specified correspondence of the sizes allows us to conclude that the diameter of the growing CNTs is determined by the particle size of a dispersed iron catalyst, similar to the above model of CNTs formation proposed by Baker [19].

Singled out on the active centers of the catalyst surface, carbon can dissolve in iron forming an implementation solid solution. The limiting solubility of carbon in *α*—iron at temperatures 600–723 °C—can be estimated by the expression [4]

4 $$ \mathrm{l}\mathrm{g}\mathrm{C}=3.22{\textstyle \hbox{-} }4509/ T{\textstyle \hbox{-} }2{.2510}^{{\textstyle \hbox{-} }4} T $$

here, *T* is temperature, K.

At a temperature of 923 K (650 °C), the maximum solubility of carbon in iron is only 0.013%. Taking into the consideration the low solubility of carbon, the migration of its atoms from active centers to the place of CNTs growth, for our opinion, is mainly due to the surface diffusion, especially that its speed by 2–3 orders is higher than internal [20]. This is also evidenced by the fact that the formed nanofibers are hollow inside. Indeed, the delivery of carbon atoms into the internal part of the fibers presupposes their diffusion through the crystal lattice of iron, i.e., internal diffusion.

The formation of ferrite and cementite (Fe_3_C) with polymorphic transformation of γ-Fe → α-Fe in the steel is the evidence of the insufficiently high internal diffusion capacity of carbon. Despite the fact that formation of graphite is energetically more favorable, the decomposition of austenite at low temperatures usually occurs with a pearlite formation [21], due to the relatively slow speed of diffusion of carbon atoms in iron. Apparently, carbon atoms in its crystal lattice have a mutual repulsion that does not accelerate carbon internal diffusion [22].

**Fig. 5:**
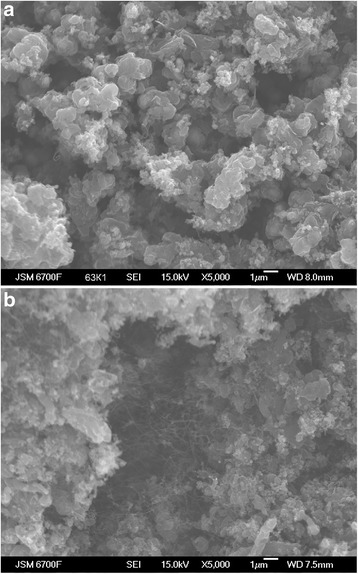
Photo of carbon nanomaterial obtained on the iron catalyst at the temperatures of 750 (**a**) and at 650 °C (**b**) material obtained on the catalyst at 750 °C

Studies have shown that the temperature of maximum rate of CNTs formation from products of air conversion of natural gas onto newly reduced iron is in the range of 600–650 °C. At temperatures above 700 °C, the obtained carbon material has a scaly shape, and despite the presence of iron (up to 13% or more), nanotubes are practically absent in it (Fig. 5a). But, nanotubes are formed if obtained at this temperature; carbon material is reprocessed by converted natural gas already at the temperature of 650 °C (Fig. 5b). This fact suggests that the mechanism of CNTs formation under these conditions proceeds through the stage of formation of iron oxides according to the reactions:

5 $$ \mathrm{F}\mathrm{e}+\mathrm{C}\mathrm{O}\to \mathrm{F}\mathrm{e}\mathrm{O}+\mathrm{C}, $$

6 $$ 3\mathrm{F}\mathrm{e}+4\mathrm{C}\mathrm{O}\to {\mathrm{Fe}}_3{\mathrm{O}}_4+4\mathrm{C}. $$

**Fig. 6:**
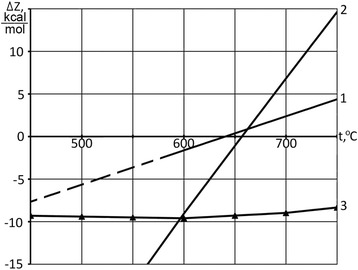
The change in the isobaric potential ∆Z from the temperature of the reactions: *1* – Fe + CO → FeO + C, P_CO_ = 0,18 atm; *2* – 3Fe + 4CO → Fe_3_O_4_ + 4C, P_CO_ = 0,18 atm; *3* – 2FeO + SiO_2_ → Fe_2_SiO_4_ ([27])

The thermodynamic possibility of these reactions in the region of moderate temperatures can be seen from Fig. 6, which shows a change in their isobaric (isobaric-isothermal) potential ∆Z (Gibbs energy) depending on the temperature. Calculations of isobaric potential of reactions are performed using the data of [23]. It should be noted for some proximity in the calculation related to the lack of thermodynamic data for CNTs formation.

As can be seen from Fig. 6, reactions (5) and (6) become thermodynamically impossible in the temperature region exceeding 700 °C. According to the experimental data, CNTs formation in this field also is terminated.

In the atmosphere of a converted natural gas, the iron oxides formed by the reactions (5) and (6) are reduced to the original metal:

7 $$ \mathrm{F}\mathrm{e}\mathrm{O}+\mathrm{C}\mathrm{O}\to \mathrm{F}\mathrm{e}+{\mathrm{CO}}_2, $$

8 $$ {\mathrm{Fe}}_3{\mathrm{O}}_4+4\mathrm{C}\mathrm{O}\to 3\mathrm{F}\mathrm{e}+4{\mathrm{CO}}_2. $$

Summing equations (5)–(8), we obtain the final gross reaction of carbon formation from carbon monoxide:

9 $$ 2\mathrm{C}\mathrm{O}={\mathrm{CO}}_2+\mathrm{C}. $$

Iron from its oxides can also be reduced in the presence of hydrogen in the gas phase according to the reactions:

10 $$ \mathrm{F}\mathrm{e}\mathrm{O}+{\mathrm{H}}_2\to \mathrm{F}\mathrm{e}+{\mathrm{H}}_2\mathrm{O}, $$

11 $$ {\mathrm{Fe}}_3{\mathrm{O}}_4+4{\mathrm{H}}_2\to 3\mathrm{F}\mathrm{e}+4{\mathrm{H}}_2\mathrm{O}. $$

Summarizing reactions (5) and (10), and also (6) and (11), we obtain the second final gross reaction of carbon formation occurring with hydrogen participation:

12 $$ \mathrm{C}\mathrm{O}+{\mathrm{H}}_2={\mathrm{H}}_2\mathrm{O}+\mathrm{C}. $$

It is obvious that the higher the content of hydrogen in the source gas, reactions (10) and (11) will run in more degrees compared with (7) and (8). This raises the proportion of carbon which forms according to the final gross reaction (12) in comparison with reaction (9) that was confirmed in the experiment [24].

The following experimentally observed fact is evidenced in a favor of an assumption about the possibility of iron oxides forming in the present conditions. In the case when the substrate for the catalyst is a plate of transformer steel, the formation of the carbon material (in any form) is suppressed. It is known that the transformer steel contains a high content of silica (up to 4.5% [25]). Silicon has a high affinity for oxygen, so even a slight presence in the gas phase of oxidants CO_2_ and H_2_O leads to its oxidation [26].

Apparently, the formed silicon oxide reacts with iron oxide (II) appearing on the active centers with the formation of chemical compounds of fayalite—Fe_2_SiO_4_. Negative value of isobaric potential of fayalite formation (line 3, Fig. 6) is evidence of the thermodynamic possibility of its formation, the data is borrowed from [27]. As a result, catalyst deactivation occurs and carbon formation is not observed. Paper [28] indicates observations in practice cases of carbon and iron oxides formation at low temperature in an environment with a high ratio of CO/CO_2_ during steel annealing in heat-treatment furnaces. Finally, iron oxides are detected in the X-ray pictures of iron catalyst samples after pre-reduction by hydrogen, and then processing by mixture of carbon monoxide with nitrogen [29].

Emitted carbon at temperatures above 700 °C has a scaly structure. While at lower temperatures, it is synthesized in the form of nanotubes. This fact suggests that in the temperature range below 700 °C, cycles of oxidation-reduction of iron catalyst play a crucial role in the process of CNTs formation. This factor, together with the inextricable relation to the process of carbon emissions by the reactions (5) and (6) contributes to the fragmentation of iron into tiny particles smaller than 300 nm and ultimately nanotube formation. At temperatures above 700 °C, decomposition of carbon monoxide occurs on another mechanism without the stage of iron oxides formation. In this case, although there is a fragmentation of the catalyst, the forming iron particles are larger and emitting carbon has a scaly shape.

It is noteworthy that the visible surface of iron particles is close to spherical under the form. Special studies [30] showed that even at room temperature, crystals of various metals under the action of surface tension forces change their form in the direction of reducing their external surface. However, at low temperatures, this process proceeds very slowly and becomes noticeable only after a long time (several months). Spheroidization of iron particles proceeds much faster at temperatures of CNTs formation and microscopic size of iron particles. Apparently, many times, repeating cycles of iron oxidation-reduction also plays a big role in the process of spheroidization which leads to a weakening of the bonds between the atoms in the crystal lattice and thereby contributes for intensification of their self-diffusion.

## Conclusions

The mechanism of occurrence and growth of CNTs on iron catalyst under conditions of low-temperature disproportionation of carbon monoxide contained in products of air conversion of natural gas is considered.

A highly dispersed structure of iron in the form of a chaotic accumulation of its particles of microscopic size is formed In terms of low-temperature reduction, the boundaries of contact of particles have the highest catalytic activity, where the nucleation of a new phase occurs in the form of ring-shaped cuffs due to emitted carbon while carbon monoxide decomposes. Fragmentation of the catalyst with separation of iron particles from the main mass occurs as the result of carbon subsequent crystallization. The outer diameter of nanotubes formed while growth of carbon cuffs is determined by the size of separated iron particles. The optimum temperature for CNTs formation from products of air conversion of natural gas is 600–650 °C. It was suggested that the mechanism of carbon nanotube formation in considered conditions flows through the intermediate stage of oxidation-reduction of iron on the active centers of the catalyst.
